# Massively parallel recordings in macaque motor cortex during an instructed delayed reach-to-grasp task

**DOI:** 10.1038/sdata.2018.55

**Published:** 2018-04-10

**Authors:** Thomas Brochier, Lyuba Zehl, Yaoyao Hao, Margaux Duret, Julia Sprenger, Michael Denker, Sonja Grün, Alexa Riehle

**Affiliations:** 1 Institut de Neurosciences de la Timone (INT), UMR 7289, CNRS-Aix Marseille Université, 13005 Marseille, France; 2 Institute of Neuroscience and Medicine (INM-6) and Institute for Advanced Simulation (IAS-6) and JARA Institute Brain Structure-Function Relationships (INM-10), 52425 Jülich, Germany; 3 Theoretical Systems Neurobiology, RWTH Aachen University, 52056 Aachen, Germany; 4 RIKEN Brain Science Institute, Wako-Shi 351-0198 Saitama, Japan

**Keywords:** Neural encoding, Motor cortex, Data publication and archiving

## Abstract

We publish two electrophysiological datasets recorded in motor cortex of two macaque monkeys during an instructed delayed reach-to-grasp task, using chronically implanted 10-by-10 Utah electrode arrays. We provide a) raw neural signals (sampled at 30 kHz), b) time stamps and spike waveforms of offline sorted single and multi units (93/49 and 156/19 SUA/MUA for the two monkeys, respectively), c) trial events and the monkey’s behavior, and d) extensive metadata hierarchically structured via the odML metadata framework (including quality assessment post-processing steps, such as trial rejections). The dataset of one monkey contains a simultaneously saved record of the local field potential (LFP) sampled at 1 kHz. To load the datasets in Python, we provide code based on the Neo data framework that produces a data structure which is annotated with relevant metadata. We complement this loading routine with an example code demonstrating how to access the data objects (e.g., raw signals) contained in such structures. For Matlab users, we provide the annotated data structures as mat files.

## Background and Summary

We publish high-dimensional and multi-scale datasets that contain recordings from motor cortex with a 10-by-10 Utah electrode array during controlled reach-to-grasp movements for two monkeys (L and N). In particular, we provide the activities of a large number of simultaneously recorded single neurons (93 and 156, for L and N respectively) along with the continuous neuronal “raw” signals (sampled at 30 kHz, and broadly band-pass filtered to 0.3 Hz–7.5 kHz). To study the local field potential (LFP)^1–3^, a down-sampled and filtered version of the latter is provided for monkey N and can be computed from the data of monkey L. These high-dimensional parallel datasets provide the opportunity for neuroscientists and computational neuroscientists to study interactions in the cortical network during different epochs of a well described behavior.

To date not many tools enable to study network coordination in high-dimensional data, because non-experimentalists, such as statisticians or theoretical/computational neuroscientists often do not have access to such data. Nevertheless, methods for correlation analysis of high-dimensional data that do not run into a combinatorial explosion and do have acceptable computing times are in high demand. This implies also the strong need for methods and tools that perform dimensionality reduction to reduce the complexity of the data and the computational load (e.g. ref. 4). The development of such analysis methods requires to know the typical features of experimental data, such as non-stationarity in time and across trials, as well as deviations from typical theoretical assumptions (e.g., Poisson distributed spike times), in order to make the methods applicable to experimental data. If these features are ignored and not considered by the method, there is a considerable danger of generating false positive outcomes and potentially wrong interpretations of the results^5,6^. The data we publish allow non-experimentalists to get insights in features of massively parallel cortical data from awake behaving animals.

Our data also provide the possibility to test and validate spike sorting methods (see ref. 7). Besides the raw data we publish the corresponding spike data resulting from an offline spike sorting using the Plexon Offline Sorter. Other sorting methods can be applied to the same (raw) data, and differences in the results can be compared and analyzed.

Complex datasets, such as those provided here (high-dimensional, multi-scale during complex behavior), are a challenge for performing reproducible analysis. Besides the often rather variable nature of the circumstances under which such data were recorded, the data additionally experience a number of often interactively performed preprocessing steps before they can be used in actual data analyses. Without a detailed knowledge about all these steps, the actual data analysis may be biased or strongly affected. In most cases, openly available electrophysiological data are not sufficiently annotated and documented in this respect. For this reason, we provide here a comprehensive description of how and under which circumstances the datasets were recorded as well as a detailed description of preprocessing steps that need to be considered before performing analyses on the data. We additionally publish a machine-readable format of these metadata including our parameters and results of the described preprocessing steps (see ref. 8 on how to create a comprehensive metadata collection in a common file format). We are aware that all this information may not be sufficient for the reproducible analysis of such data. The reason is that reproducible workflows including the provenance trail are not yet established for electrophysiological neuroscience, especially not for such complex experiments as presented here. Thus, we hope to make researchers from other fields, such as computer science, process engineering or others become interested to support the neuroscience field with solutions for reproducible research. Existing approaches from neuroinformatics / computer science are not yet sufficient to generate a complete provenance track of the processes involved (see e.g., ref. 9). We provide here a concrete use case for such a development.

In summary, we publish two datasets (one for each monkey, both having performed the same behavioral task) containing the raw neuronal data, offline sorted single and multi unit activity, the behavioral data, and metadata containing all information about each dataset including our parameters and results of the described preprocessing steps (Data Citation 1). The collected information are partly taken from^8,10–12^. These are the first published datasets of massively parallel recordings from monkeys while they perform a complex instructed delayed reach-to-grasp task.

## Methods

### Subjects

All animal procedures were approved by the local ethical committee (C2EA 71; authorization A1/10/12) and conformed to the European and French government regulations. Monkey L (female) and N (male) are both Rhesus macaque monkeys (*Macaca mulatta*) which were trained to perform an instructed delay reach-to-grasp task for food reward (drops of apple sauce). Generally, the training started with getting the monkey accustomed to the experimenter and the monkey chair. After this setting-in period, the monkey had to learn a complex reach-to-grasp task in which she/he had to control both the grip type used to grasp an object and the force required to displace it. The training of the monkey was completed after she/he was able to perform the correct grip in 85% of the trials on average.

### Monkey L

Monkey L was born on March 15, 2004. She started training in a preliminary version of the task in 2008. After a long break in 2009, she restarted training in June 2010 on the final version of the task. She had one phalanx missing on her right thumb and was therefore trained to perform the task with the left hand. The training was easy and fast with monkey L. She habituated rapidly to the training chair and could start the task training within a month from chair training onset. Generally, monkey L was described as being eager to work, quick and efficient during the task, but rather nervous. The surgery for the Utah Array implant took place on September 15, 2010 (see Sec. ‘Surgery Array Location’). About 2 weeks after the surgery, the recordings started. At that time she had a body weight of 5 kg. Neuronal data were recorded during task performance until May 6, 2011. After this date, the quality of the recordings deteriorated suddenly and did not recover. On June 23, 2011, during a short surgery, the array wire bundle was cut, the connector was removed, but the array left in place. Monkey L is still alive. File names from monkey L are identified by the letter “l”.

### Monkey N

Monkey N was born on May 15, 2008. He was initially trained to perform the task with the right hand between April 2012 and April 2013. After a first and disappointing recording period from the left hemisphere which ended in September 2013, he was retrained to perform the task with the left hand and implanted with another Utah Array in the right hemisphere on May 22, 2014 (see Sec. ‘Surgery and Array Location’). In general, he was calmer than monkey L, but overall less motivated. He learned the task at a much slower pace, performed on average less trials per recording and was often less attentive than monkey L. When the recording period started he had a body weight of 7 kg. Neuronal data were recorded until January, 2015. The quality of the recordings decayed gradually throughout the recording period. At the end of February, 2015, the monkey damaged the wire bundle between the connector and the array. The wire bundle was then cut and the connector was removed, but, as for monkey L, the array was left in place. Monkey N is also still alive. File names from monkey N are identified by the letter “i” (second letter of monkey N’s name, as reference for the recordings obtained from the second hemisphere).

### Surgery and Array Location

At the end of the training period, each monkey was chronically implanted with a Utah array (Blackrock Microsystems, Salt Lake City, UT, USA) in the motor cortex contralateral to the working hand. The array consisted of one 10-by-10 electrode grid with 96 active iridium oxide electrodes. Each electrode was 1.5 mm long with an inter-electrode distance of 400 *μ*m. The electrodes had on average an impedance of 50 kΩ measured at 1 kHz (impedance values were provided by Blackrock Microsystems for each electrode). The impedance of each electrode is indicated in the respective metadata file for each dataset. The electrodes were connected through a wire bundle of 4 cm length to a high-density CerePort Connector (Blackrock Microsystems). In parallel, two reference/ground wires were connected to the connector pedestal. The surgery was performed under deep general anesthesia using full aseptic procedures. Anesthesia was induced with 10 mg/kg intramuscular ketamine and maintained with 2-2.5% isoflurane in 40:60 O2 air. To prevent cortical swelling, 2 ml/kg of intravenous mannitol infusion was slowly injected over a period of 10 min. A large craniotomy (20×20 mm and 30×20 mm for monkey L and N, respectively) was performed over the motor cortex and the dura was incised and reflected. The array was inserted using a pneumatic inserter (Array Inserter, Blackrock Microsystems) and covered with a sheet of an artificial non-absorbable dura (Preclude, Gore-tex) to avoid attachment of the array to the dura. The two reference/ground wires were then placed underneath the dura onto the cortical surface. The real dura was sutured back. The dura was then also covered with a piece of an artificial absorbable dura (Seamdura, Codman). The bone flap was put back at its original position and attached to the skull by means of a 4×40 mm strip of titanium (Bioplate, Codman). The connector pedestal was fixed to the skull on the opposite side with titanium bone screws (Bioplate, Codman). The skin was sutured back over the bone flap and around the connector. The monkey received a full course of antibiotic (Baytril and Marbocyl for monkey L and N, respectively), anti-inflammatory (Tolfedine and Ketofen for monkey L and N, respectively) and analgesic (Vetergesic) before returning to the home cage and on the following days up to complete recovery (ref. 10).

**Figure 1:**
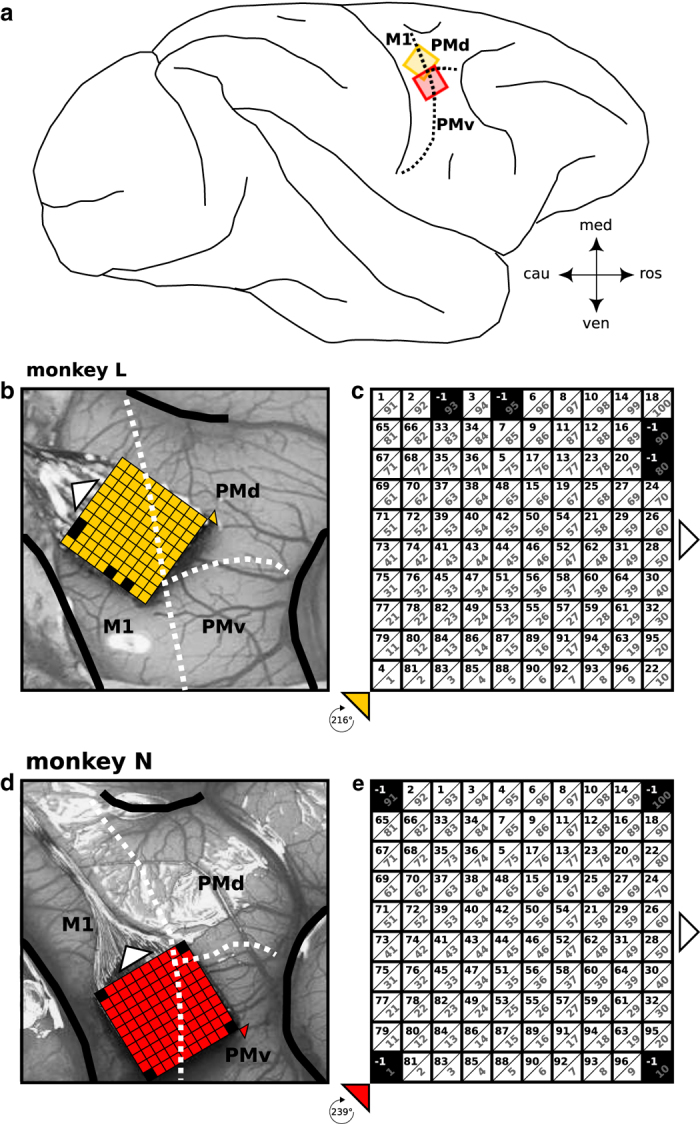
Implant locations of the Utah arrays. The figure displays the anatomical location of the Utah Array of both monkeys after implantation as well as the fabrication settings of each array provided by Blackrock. (**a**) Schematic drawing of a macaque cortex with implant location of the array of both monkeys. Both arrays were implanted along the central sulcus and overlapping the putative border (dotted line) between primary motor cortex (M1) and dorsal or ventral premotor cortex (PMd or PMv) of the right hemisphere. (**b**, **d**) Exact location of the array for each monkey in the close-up picture of the implantation site taken during the surgery (length of an array side is 4 mm). The central sulcus, the arcuate sulcus and the superior precentral dimple are emphasized as thick black lines (to the left, right and top, respectively). (**c**, **e**) Scheme of each array in a default array orientation where the wire bundle (indicated with white triangles in (**b**-**e**)) to the connector points to the right. Each array scheme shows the 10-by-10 electrode grid with the electrode identification numbers (IDs) defined by Blackrock (black numbers) and the location of the non-active electrodes (indicated in black as ID=−1). Gray numbers show an alternative set of connector-aligned electrode IDs, assigned based on electrode location with respect to the connector of the array, which are more practical for data analysis and comparisons across monkeys. In order to best cover the arm/hand representation of the primary motor cortex, each array was rotated for the implantation. The center of rotation is indicated by a colored triangle (**b**-**e**), stating below (in **c** and **e**) the degree of rotation for each array.

In both monkeys, the array was implanted a few millimeters anterior to the central sulcus with the wire bundle pointing in medio-caudal direction. We aimed at implanting the array in the arm/hand representation of the primary motor cortex with the most anterior electrodes encroaching upon the premotor cortex. For this reason, the arrays were rotated by 216 and 239 degrees for monkey L and N, respectively (cf. Fig. 1b-e). The center of rotation was set to the bottom left corner of the default array orientation where the wire bundle to the connector points to the right (cf. Fig. 1c,e). With respect to the precentral dimple and the spur of the arcuate sulcus, the array was located a few millimeters more lateral in monkey N than in monkey L (see Fig. 1). Therefore, with respect to putative borders between MI, PMd, and PMv^13^, the anterior electrodes of the array are assumed to cover part of PMd in monkey L and part of PMv in monkey N.

### Daily Routines

Recordings were performed on regular workdays. Weekends as well as holidays were usually excluded. Both animals were never fluid restricted. However during workdays, they were exclusively fed with dry food when returned to the home cage at the end of the recording session. The amount of dry food was controlled to ensure weight stability over the course of the experiment. During the weekend and during holidays the food was supplemented with fruits and vegetables. A typical recording day consisted in: a) taking the monkey out of the cage, b) placing him/her in front of the experimental apparatus (see Sec. ‘Experimental apparatus’) in the primate chair, c) conducting several recording sessions while the monkey performed the behavioral task, and then d) returning the monkey back to its cage. A single recording session lasted between 10 to 20 min. The number of recording sessions per day depended on the motivation of the monkey, but on average a recording day lasted for 1.5 h.

### Task

**Figure 2:**
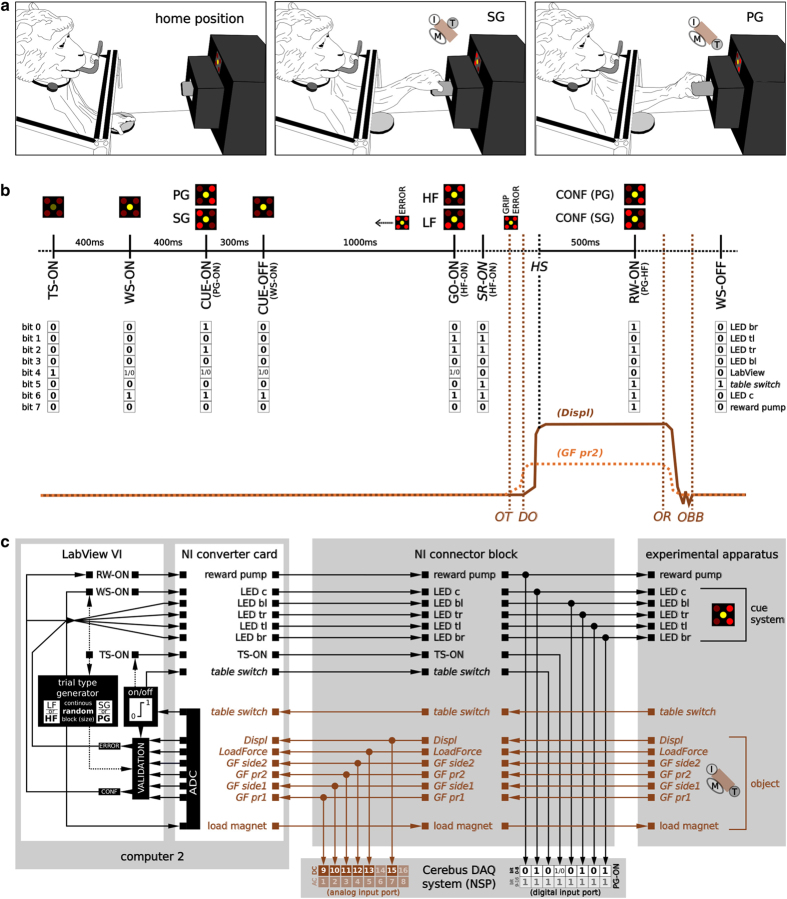
Overview of the experimental apparatus and behavioral control system. (**a**) Sketches of the experimental apparatus and the monkey performing the reach-to-grasp task. Left: monkey in its home position with the working hand on the table switch. Middle and right: monkey grasping the object with a side grip (SG) and a precision grip (PG), respectively. Insets of middle and right sketch show the actual position of the index finger (I), the thumb (T), and the middle finger (M) on the object (brown cube)^11^. (**b**) Trial scheme of an example trial with the respective visual cues (different illumination combinations of the 5 LEDs illustrated on top) shown to the monkey at its respective times. The behavioral events are marked along the time axis (see main text for abbreviations). Events with black font mark digitally recorded events, whereas events with brown font indicate events (object touch OT, object release OR, displacement onset DO, and object back to baseline OBB) which were extracted offline from baseline deviations of the analog signals of the object’s sensors. Additionally, we indicate by italic fonts events which were generated by the monkey, while all other events are produced by LabView. The 8-bit binary code for the digital event signals sent from LabView VI to the NSP at the respective times is shown below the time axis. Example traces for the analog signals of the HE sensor (Displ; dark solid line) and one of the 4 FSR sensors located at the object’s surface (GF pr2, light dotted line) used to monitor the monkeys behavior and extract OT, OR, DO, and OBB are shown at the bottom. (**c**) Outline of the devices and their wiring controlling the behavior. All analog signal streams are colored in brown, whereas all digital signal streams are colored in black.

During a trial, the monkey had to grasp the object using either a side grip (SG) or a precision grip (PG). The PG had to be performed by placing the tips of index and thumb in a groove on the upper and lower sides of a cubic object, respectively (see Fig. 2, right; ref. 11). For SG, the tip of the thumb and the lateral surface of the other fingers were placed on the right and left sides of the object (see Fig. 2a, middle; ref. 11). The monkey had to pull the object towards him/her against one of two possible loads requiring either a high or low pulling force (HF and LF, respectively). As a result, from the possible combinations of grip types and object loads, the monkey had to perform in total four different trial types (SG-LF, SG-HF, PG-LF, PG-HF). In each trial, the grip and force instructions for the requested trial type were provided to the monkeys independently through two consecutive visual cues (CUE and GO) which were separated by a one second delay. Both cues were coded by the illumination of specific combinations of two LEDs of a five-LED cue panel positioned above the target object. Details on how the task, the trial scheme and the corresponding behavior of the monkey were controlled are stated in Sec. ‘Behavioral control system’.

### Setup

**Figure 3:**
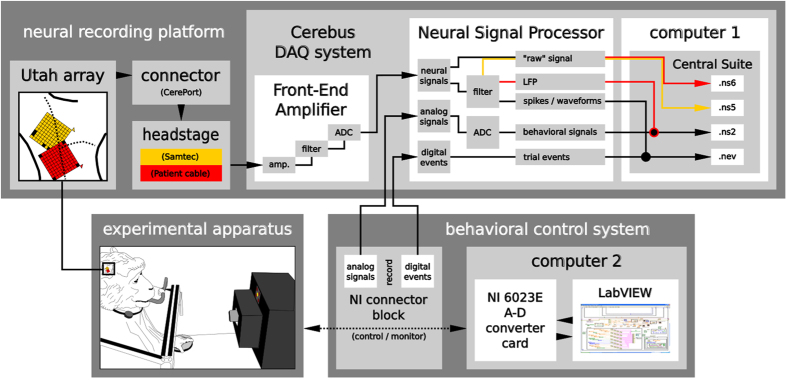
Overview of the setup. The setup consisted of three main parts: the neural recording platform, the experimental apparatus, and the behavioral control system. The neural recording platform (top) was composed of the implanted Utah array with its corresponding connector (CerePort), a headstage (Samtec or Patient cable), and the Cerebus data acquisition (DAQ) system (i.e. the Front-End Amplifier, Neural Signal Processor (NSP), and the Cerebus control software, Central Suite, installed on the setup computer 1). The experimental apparatus (bottom left) consisted of the physical devices which the monkeys had to interact with (i.e., the visual cue panel (square with 5 LEDs), the target object, the table switch, and the reward system). The behavioral control system (bottom right) was built from hard- and software of National Instruments (NI, National Instruments Corporation, Austin, Texas, USA). It was composed of a NI connector block which was linked via a NI 6023E A-D converter card to setup computer 2 on which the NI system design software, LabView, was running. To record the behavioral data the behavioral control system was interlinked with the neuronal recording platform via the NSP and the NI connector block. All three parts are separately described in more detail in the main text, as well as Figures 2 and 4. Setup differences between the two monkeys are indicated in yellow and red for monkeys L and N, respectively.

The setup was organized in three major parts, the neural recording platform, the experimental apparatus, and the behavioral control system. The components of each part and their connections are summarized in Fig. 3 and described in more detail in the following. Even though overall the same setup was used for both monkeys, it differed in a few aspects which are described in more detail below and, in the referenced figures, marked in yellow and red for monkey L and N, respectively.

### Experimental apparatus

The experimental apparatus was composed of a table switch, a target object, a visual cue, and a reward system. On each recording day, the monkey was seated in a custom-made primate chair and placed in front of that apparatus. The non-working arm of the monkey was loosely restrained in a semi-flexed position.

The table switch was installed close to the monkey at waist level, 5 cm lateral to the mid-line of the apparatus. To control the home position of the working hand, the monkey had to press down this table switch between each reach-to-grasp movement.

The target object was a stainless steel rectangular cuboid (40×16×10 mm) pointing towards the monkey and rotated 45 degrees around the depth axis from the monkey’s point of view (Fig. 2a). It was located 13 cm away from the table switch at 14 cm height. The posterior end of the object was attached through a low-friction horizontal shuttle to a counterweight hidden inside the apparatus, which was used to set the object load. The object load was set to one of two possible values to define the force type (LF and HF) needed for pulling the object in each trial by deactivating and activating an electromagnetic weight resting below the counterweight inside the apparatus. When activated, it attached to the counterweight and increased overall weight from 100 g to 200 g, which corresponds roughly to a pulling force of 1 N and 2 N for LF and HF, respectively.

As already mentioned, the object was equipped with six sensors which monitored the monkey’s reach-to-grasp behavior: five force sensitive sensors (FSR sensors), and one hall-effect sensor (HE sensor). The four FSR sensors located on the object surface provided continuous measurement of the grip forces applied on the object sides by the index and middle finger, as well as the thumb. The different activation patterns of these four FSR sensors, in particular the different placement of the thumb (see Fig. 2a), were used to detect online if the correct grip type was performed. The fifth FSR sensor was installed between the object and its counterweight. This FSR sensor was used to measure the horizontally applied force needed to oppose the corresponding object load. Due to the low, but still existing friction of the object moving inside the horizontal shuttle, the measured force signal of this sensor is not perfectly proportional to the horizontal force needed to lift the opposed object load, but sufficient to distinguish between LF and HF settings. The horizontal displacement of the object over a maximal distance of 15 mm was measured by the HE sensor. All sensors of the object are summarized in Table 1.

The visual cue system, composed of a square of five LEDs (size 10×10 mm), was located just above the target object and used to instruct the monkey about the requested behavior. While the central yellow LED was used to warn the monkey that a trial had started, the four red corner LEDs were used to code separately the grip and the force type for the requested trial type of each trial. In this context the illumination of the two left, the two right, the two bottom, or the two top LEDs coded for SG, PG, LF, or HF, respectively (see Fig. 2b for illustration).

The reward system consisted of a bucket filled with apple sauce and equipped with a feeding tube and a pump allowing to deliver on demand the reward (few drops of the apple sauce) to the monkey (Fig. 2a).

### Behavioral control system

The core of the behavioral control system is a custom-made Virtual Instrument (VI) in LabView that controls the digital event sequence and the requested behavior of each trial in a recording. A digital event reflects hereby the activation or deactivation of a physical device of the experimental apparatus. In this context, the LabView VI is responsible to activate and deactivate the LEDs of the visual cue system, the reward pump, and the electromagnet. The latter is not controlled by a digital event, but by an analog square signal that switches the magnet on or off. To control the requested behavior, the LabView VI monitors the monkey’s manipulation of the table switch and the target object. The table switch as well as all sensors of the target object produce continuous analog signals that are digitized by the NI converter card and fed into the LabView VI of the setup computer (see Fig. 3, computer 2). The square signal of the table switch is then reinterpreted online as digital activation or deactivation event. Fig. 2c displays a schematic diagram on how the physical devices of the experimental apparatus are connected to the setup computer and controlled and monitored by the LabView VI. We will now describe a typical execution of the LabView VI during a recording session in more detail.

Each single trial was run and controlled as follows: The LabView VI only started a trial when the monkey deactivated the table switch by pressing and holding it down (home position, Fig. 2a, left). This required not much muscle activity, but simply the weight of the monkey’s hand on top of the smooth-running switch. If the table switch was deactivated, the LabView VI internally initiated a trial (TS-ON). In parallel, the program picked randomly with equal probability one of the possible trial types (SG-LF, SG-HF, PG-LF, or PG-HF) and activated or deactivated the electromagnet accordingly to fit the chosen load force of the object (e.g., activated for HF). To inform (or warn) the monkey that a new trial has started, 400 ms after the trial was initiated by the program (i.e., TS-ON) the central LED was illuminated (WS-ON). Then, 400 ms after WS-ON the grip type was revealed to the monkey by illuminating the corresponding corner LEDs of the chosen trial type (CUE-ON, e.g., left LEDs for SG-ON). The LEDs of this first cue were turned off again after 300 ms (CUE-OFF). The CUE-OFF was followed by a 1000 ms preparatory delay at the end of which the monkey was informed about the upcoming force type by again illuminating the corresponding corner LEDs of the chosen trial type (GO-ON, e.g., top LEDs for HF-ON). This second cue also served as a GO signal for the monkey to initiate the movement which was registered by the activation of the table switch (SR-ON) when the monkey released it after a variable reaction time (RT). The execution of the movement was composed of reaching, grasping, pulling and holding the object in the position window for 500 ms. The LabView VI controlled the movement execution online by checking the used grip type, the object displacement and the hold time. For checking the grip type, the grasp of the object was registered by small deflections of the FSR surface sensor signals caused by the monkey’s fingers. An FSR sensor was registered as activated if the deflection surpassed a predefined threshold. The pattern of activated FSR sensors was then used by the LabView VI to monitor if the monkey performed the requested grip type. This meant, in particular, to check for SG and PG, if the FSR sensor on the right (GF side2), or on the bottom (GF pr2) of the object was activated by the monkey’s thumb, respectively (see Fig. 2a, middle and right). The other two sensors that measured force from the index and middle fingers for the two grip types (GF side1, and GF pr1) were not controlled online. If the correct grip was detected, the grip cue was illuminated again as a positive feedback. To check the object displacement, the LabView VI measured if the deflection of the HE sensor signal of the object was within the two defined position thresholds (4 mm and 14 mm). The time point at which the displacement signal surpassed the lower threshold was used by the LabView VI to define the estimated start of the holding period (HS) online. If the object remained within the position window for 500 ms after the HS was set, LabView activated the reward pump which provided the monkey with a drop of apple sauce as reward for a successful trial. The time until the reward pump was deactivated again by LabView was proportional to the duration of the object hold in the position window, with a maximum duration (i.e., a maximum amount of reward) for a 500 ms holding period. With this mechanism, both monkeys rapidly learned to hold the object at least 500 ms in nearly all trials. In parallel to the deactivation of the reward pump, LabView turned off all LEDs to indicate that the running trial ended (WS-OFF). The monkey was allowed to release the object at its own pace as soon as it received the reward. A new trial sequence (TS-ON) was started by LabView as soon as the monkey returned to the home position (new deactivation of the table switch).

An abort of the described trial sequence by LabView (error trial) was triggered by the following three scenarios: (i) the monkey released the table switch before the GO cue, (ii) the wrong grip type was registered, and (iii) the object was not pulled in the position window. In case one of these scenarios was registered by LabView the trial was aborted. For monkey L, the LabView VI provided additionally a negative feedback when aborting a trial by flickering all LEDs three times.

**Figure 4:**
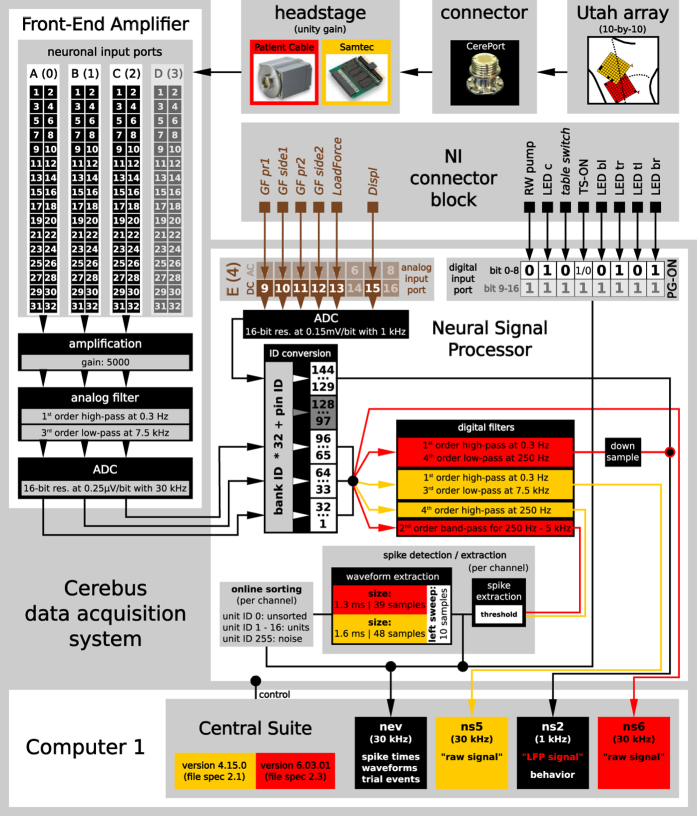
Sketch of the components related to the recording of the neuronal signals. Data were recorded using a Utah array, which was linked via its connector (CerePort) to a headstage (Samtec or Patient Cable) with a unity gain. From there the neural signals were transfered to the Cerebus Front-End Amplifier, where they were amplified, filtered and digitized. The digitized signals were converted into a single multiplex optical output and sent via a fiber-optic data link to the Neural Signal Processor (NSP), which is controlled by the Cerebus control software (Central Suite). Within the NSP the time points and waveforms of potential spikes were extracted online from a correspondingly processed copy of the neural signals and saved in the nev file. Simultaneously, the continuous raw signals (sampled at 30 kHz) were saved in the ns5 (for monkey L) or ns6 file (for monkey N). In parallel to the neural signals the NSP received also the digital trial events produced by the LabView VI, and the analog signals of the object’s sensors via the NI connector block of the behavioral control system. While the digital trial events were saved along with the extracted potential spikes in the nev file, the analog signals of the sensors were digitized and saved in the ns2 file. For monkey N, a filtered and downsampled version of the neural signals (0.3–250 Hz at 1 kHz) was also saved in the ns2 file. Components and settings specific to monkey L and N are indicated by yellow and red, respectively.

As displayed in Fig. 2c, the behavioral control system was connected to the NSP of the Cerebus DAQ system to store the trial event sequence and the monkey’s behavior of each trial in a recording along with the neural data registered by the neural recording platform. For this, the analog signals of the sensors of the target object were copied from the NI connector block to the analog input port of the Cerebus System NSP via DC coupled BNC cables and connectors. In the NSP they were digitized with a 16-bit resolution at 0.15 mV/bit and a sampling rate of 1 kHz and saved in the ns2 file under the channels ids listed in Table 1 (see Sec.‘Data-records’). All digital or digitized events that register the activation and deactivation of the table switch, the LEDs of the cue system, and the reward pump, as well as the internally generated digital trial start event (TS-ON) were coded as a 8-bit binary signal (see Table 2) and transferred via the NI connector block to a 16-bit DB-37 input port of the NSP where they occupy the first 8 digits (remaining digits are set to 1). In the NSP the now 16-bit binary signal of each event was stored in its decimal representation and with its corresponding time point in the nev file (see Table 2 and Fig. 4).

### Neural recording platform

The recording of the neural signals was performed using a neural recording platform with components produced by Blackrock Microsystems (Salt Lake City, UT, USA, www.blackrockmicro.com). The platform consisted of the multi-electrode Utah array, a headstage, and a Cerebus data acquisition (DAQ) system. The latter is composed of a Front-End Amplifier, a real-time Neural Signal Processor (NSP) and the control software, Central Suite (version 4.15.0 and 6.03.01 for L and N, respectively), running on Windows XP for L, and Windows 7 for N on the setup computer 1 (see Fig. 4). The Cerebus DAQ system was also connected to the behavioral control system via the NI connector block to save the analog behavioral data and digital trial event signals that were described in the previous section in parallel with the neural signals. All data were transmitted from the NSP via an ethernet cable to be saved first locally on the setup computer 1. After a recording day, all recordings were transferred to a data server. In the following, we will describe the function of the different components of the neural recording platform in more detail.

The implant location of the Utah array, as well as the electrode configuration of the array of each monkey was described previously (see Sec. ‘Surgery-Array location’ and Fig. 1). The electrode identification numbers (IDs) are determined by how the electrodes of the array are wired and connected to the Cerebus Front-End Amplifier (see below).

The analog Blackrock headstage with unity gain (Samtec for monkey L, and Patient Cable for monkey N) was used to reduce the environmental noise. Overall, the reduction of the noise was better with the Patient Cable than with the Samtec headstage.

In the Front-End Amplifier, each of the 96 neural signals was differentially amplified with respect to the reference input of its corresponding connector bank (gain 5000) and filtered with a 1st-order 0.3 Hz high pass filter (full-bandwidth mode) and a 3rd-order 7.5 kHz Butterworth low pass filter. After that, the band-pass filtered neuronal signals were digitized with 16-bit resolution at 0.25 V/bit and a sampling rate of 30 kHz, in the following called “raw signal”. The digitized signals were converted into a single multiplexed optical output and transmitted via a fiber-optic data link to the NSP. In the NSP the raw signals were saved in an ns5 file for monkey L and in an ns6 file for monkey N. The file format depended on the firmware and software version of the Cerebus DAQ system. In addition to the neural signals, the NSP received the analog behavioral signal recorded by the behavioral control system via the analog input port. These behavioral signals were digitized and saved with a sampling rate of 1 kHz in an ns2 file. For monkey N, the ns2 file also contained a filtered and downsampled version of the raw signals, in the following called “LFP data”. To extract the LFP data, a copy of the raw data was online digitally low-pass filtered at 250 Hz (Butterworth, 4th order), and downsampled to 1 kHz within the NSP.

The NSP performed also an online spike waveform detection and classification controlled via the Central Suite software. The sorted spikes were used for a first online inspection of the data as well as for selecting and saving the spike waveforms for offline sorting. For this purpose the neuronal raw signals were for monkey L online high-pass filtered at 250 Hz (Butterworth, 4th order) and for monkey N band-pass filtered between 250 Hz and 5 kHz (Butterworth, 2nd order). Afterwards, the waveforms were detected by threshold crossing (manually set). These waveforms were then sorted by requesting the signal from identified neurons to follow through up to five hoops set by the user (all individually for each channel). To get an overview of the quality of the data during the recordings, the sorted waveforms were displayed in the online classification window provided by Central Suite.

The thresholds (one for each channel) for the spike waveform detection were not modified during a session and were saved in the nev file for each session along with all other settings (e.g., filter settings) and configurations of Central Suite. The data and corresponding settings of Central Suite can also be inspected offline using the Blackrock software CentralPlay even in the absence of the Blackrock hardware system. Each time the high-pass filtered signal passed the threshold, a snippet of 48 samples (1.6 ms) for monkey L and 38 samples (1.3 ms) for monkey N was cut and saved as potential spike waveform. The snippet was cut with 10 sample points before threshold crossing and 38 or 28 points after for monkey L or N, respectively. Waveforms identified as potential single units (online sorted spikes) were labeled with IDs from 1 to 16. Unsorted waveforms were labeled with ID 0. These potential spike waveforms were saved together with their respective time stamps in the nev file. Due to the high number of electrodes, online spike-sorting was moderately reliable. We therefore decided to re-sort spiking activity offline on each channel using the Plexon Offline Spike Sorter (Plexon Inc, Dallas, Texas, USA, version 3.3, for details see Sec. Data Preprocessing) based on the on-line detected threshold crossing events (i.e., not based on a new thresholding of the raw data). Results of offline sorting were saved in a copy of the original nev file with an updated file name.

In the following we describe how the numeric channel IDs relate to the setup. The neuronal signal inputs to the Front-End Amplifier were grouped into four banks (A-D or 0-3) from which only the first 3 were used. Each bank consists of a male header with 34 pins of which 32 were the neuronal signal input channels. The other two channels served as reference and ground, respectively. In Central Suite, the identification (ID) number of each electrode of the array is defined by the position on the input bank and pin of the Cerebus Front-End Amplifier. For this Central Suite multiplies the bank ID (0, 1, 2, or 3) with the number of pins for neural signal input channels (32) and adds the ID of the pin the electrode is connected to (cf. ID conversion in Fig. 4). The electrode wiring of the Utah array is, though, not coordinated to the input banks of the Front-End Amplifier which leads to spatially unordered electrode IDs.

Nevertheless, Utah arrays are fabricated usually in the same way where the corner electrodes are unconnected. In this case the default electrode ID configuration is unordered, but usually the same for different arrays (cf. electrode configuration of monkey N in Fig. 1). On request, as it was done for monkey L, the unconnected electrodes may be on different places on the array. This allows one to better control the orientation of the array on the cortical surface. This led to the different ID sequences of the arrays for monkey L and N (cf. Fig. 1). To facilitate the comparison of results between arrays with different electrode configurations, we assigned new IDs that reflect the spatial organization of the array. For this we used as reference the lower left corner electrode, when the wire bundle to the connector is showing to the right. These fabrication-independent, connector-aligned array IDs increase linearly from bottom left (ID 1) to top right (ID 100), row by row. They are also shown in Fig. 1d as gray numbers in the array sketch, which thereby provides the mapping of the introduced connector-aligned IDs to the original IDs assigned by the data acquisition system of Blackrock Microsystems.

**Figure 5:**
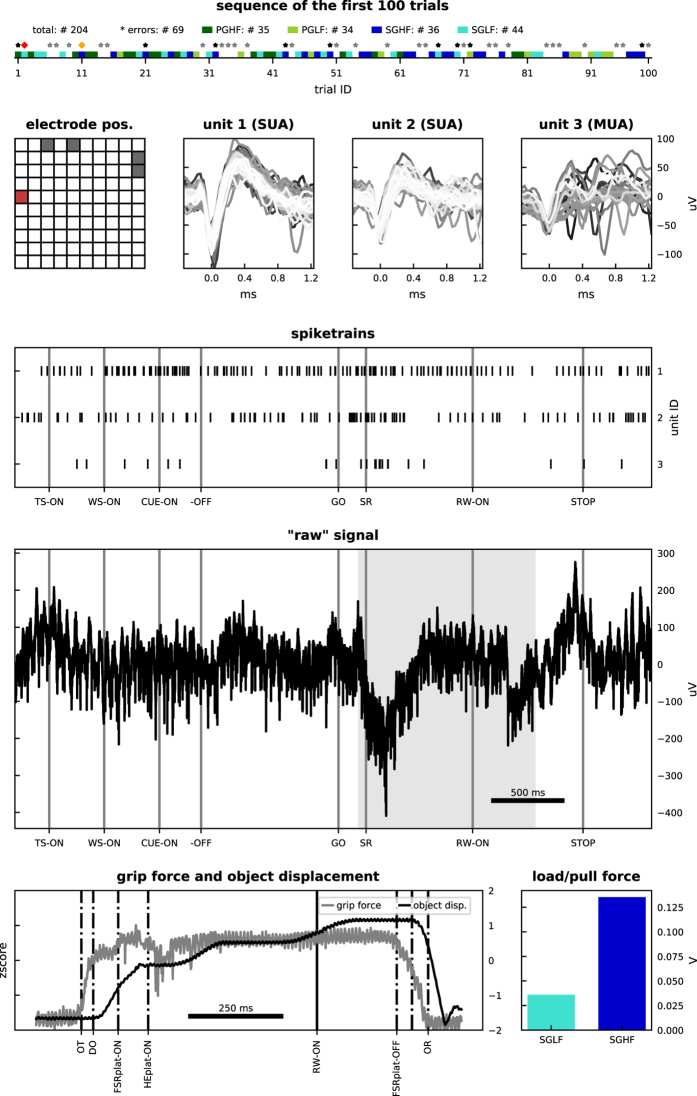
Overview of data types contained in l101210-001. The figure displays the different data types contained in the selected dataset of monkey L. Top panel: sequence of the first 100 trials (for trial types and errors see color in legend) and the total number of trials (see # for correct, error, trial types in legend); the red diamond marks the selected trial (trial ID: 2) for panels below; the orange diamond marks an additional trial selected to demonstrate load/pull force differences between the averaged load force signals in the bottom right panel. Asterisks indicate error trials (black asterisks: grip errors). Second row, left panel: position of selected electrode (in red) for the data plots (electrode ID: 71). Second row, remaining panels: waveforms of three units from the selected electrode. Third row: spike trains of displayed units for the selected trial. Forth row: raw signal for the selected trial; gray shaded area marks the time window corresponding to the bottom left panel. Bottom left panel: grip force (gray) and object displacement (black) signals for the selected trial. Bottom right panel: averaged load/pull force signals for the duration of the plateau of the grip force signal for the selected LF and HF trial. Important trial events are indicated as vertical lines in the corresponding data plots.

**Figure 6:**
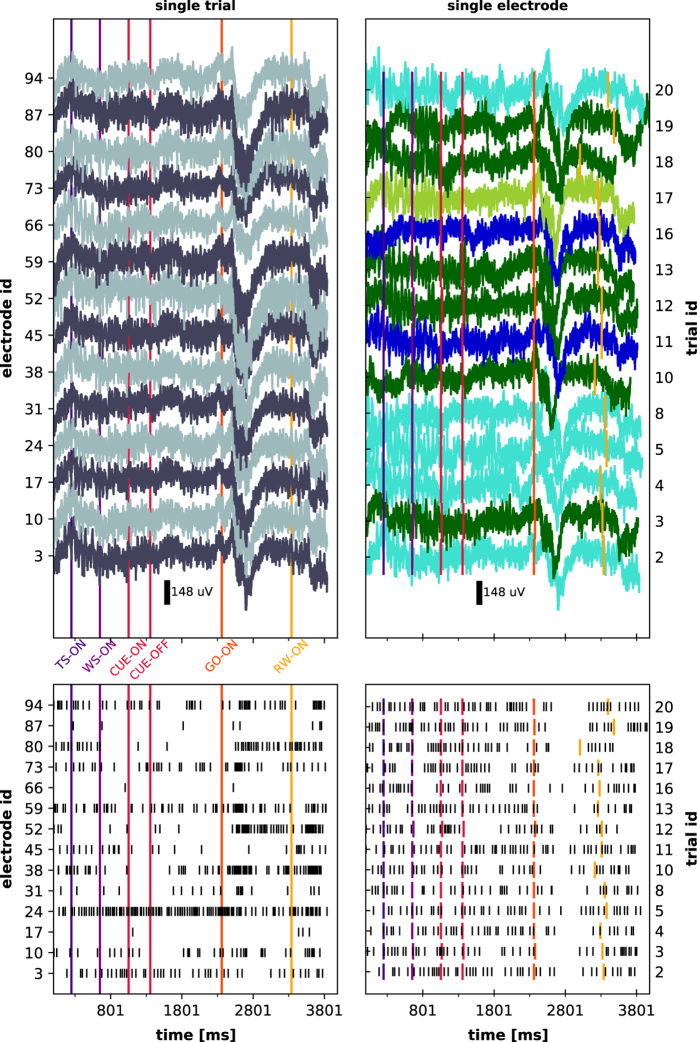
Overview of raw signal and spike data of monkey L (l101210-001). Left panels: Raw signal (top) and spike data of unit IDs 1 on each given electrode (bottom) for a single trial (trial ID: 2) across a selection of electrodes. Right panels: Raw signal (top) and spike data from single unit ID 1 (bottom) across selected correctly performed trials on one electrode (electrode ID: 3). Trial events (TS-ON, WS-ON, CUE-ON, CUE-OFF, GO-ON, and RW-ON) are indicated as colored vertical lines in each plot. Trial types of selected trials in upper right panels are indicated as color (SGHF: dark blue; SGLF: cyan; PGHF: dark green; PGLF: light green).

**Figure 7:**
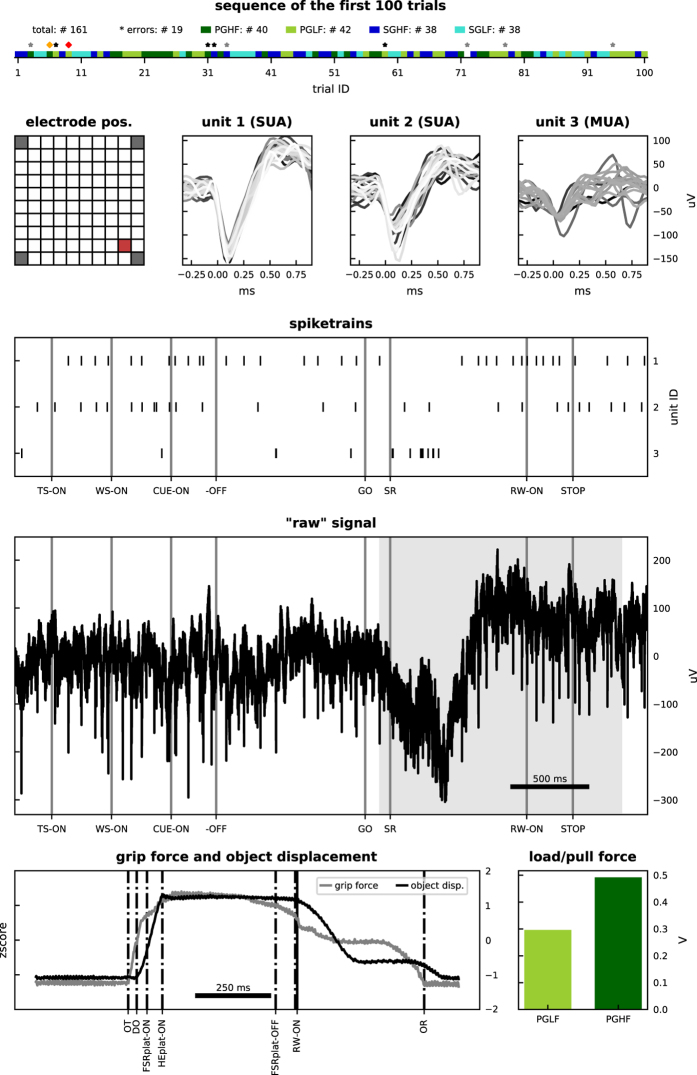
Overview of data types contained in i140703-001. The figure displays the different data types contained in the selected dataset of monkey N. Top panel: sequence of the first 100 trials (for trial types and errors see color in legend) and the total number of trials (see # for correct, error, trial types in legend); the red diamond marks the selected trial (trial ID: 9) for panels below; the orange diamond marks an additional trial selected to demonstrate load/pull force differences between the averaged load force signals in the bottom right panel. Asterisks indicate error trials (black asterisks: grip errors). Second row, left panel: position of selected electrode (in red) for the data plots (electrode ID: 63). Second row, remaining panels: waveforms of three units from the selected electrode. Third row: spike trains of displayed units for the selected trial. Forth row panel: raw signal for the selected trial; gray shaded area marks the time window corresponding to the bottom left panel. Bottom left panel: grip force (gray) and object displacement (black) signals for the selected trial. Bottom right panel: averaged load/pull force signals for the duration of the plateau of the grip force signal for the selected LF and HF trial. Important trial events are indicated as vertical lines in the corresponding data plots.

**Figure 8:**
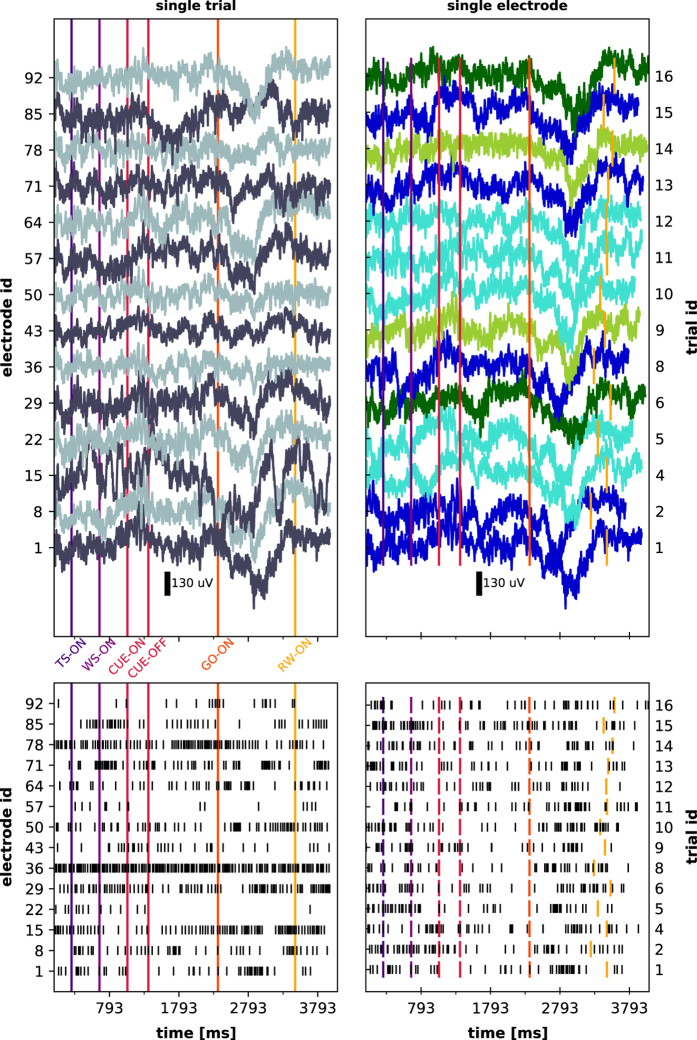
Overview of LFP and spike data of monkey N (i140703-001). Left panels: LFP data (top) and spike data of unit IDs 1 on each given electrode (bottom) for a single trial (trial ID: 1) across a selection of electrodes. Right panels: LFP data (top) and spike data from single unit ID 1 (bottom) across selected correctly performed trials on one electrode (electrode ID: 1). Trial events (TS-ON, WS-ON,CUE-ON, CUE-OFF, GO-ON, and RW-ON) are indicated as colored vertical lines in each plot. Trial types of selected trials in upper right panels are indicated as color (SGHF: dark blue; SGLF: cyan; PGHF: dark green; PGLF: light green).

A visual summary of the available data is given in Fig. 5 and Fig. 6 for monkey L, and Fig. 7 and Fig. 8 for monkey N. The first of these figures shows the sequence of trials as well as selected raw recorded time series, spike trains, unit wave forms, and behavioral signals for one particular trial. The second of these figures contrasts parallel neuronal data across channels in a specific trial with neuronal data across trials in a specific channel.

### Data Preprocessing

After the recordings, a number of preprocessing steps (*pre* in the sense of before the actual upcoming data analysis, but being the post-processing after the recording) were performed as described below. This includes (i) the translation of the digital events from their binary codes set by the DAQ system to a human-readable format putting the events in context of the expected executed trial event sequence, (ii) the offline detection of behavioral trial events and the offline inspection of the object load force from the analog signals recorded by the sensors of the target object, and (iii) the offline spike sorting.

### Translation of digital events to trial events

Table 2 lists the 8-bit combinations used by LabView to encode or control the status of the different hardware parts of the Experimental Apparatus. During the experiment, these 8-bit combinations were sent in parallel to the NSP of the Cerebus DAQ system, converted to decimal event codes and saved along with their time stamps in the nev file. In a first preprocessing step, these event codes needed to be translated to a human-readable format and put into context of an expected trial event sequence. The validation against the latter was used to identify incomplete, correct and error trials. Error trials were further differentiated into error types (e.g., grip error). The translation and interpretation of digital events (cf. Table 2) is performed automatically by the provided code to load data (see Sec. ‘Usage Notes’) and is additionally saved as part of the metadata.

### Preprocessing of behavioral analog signals

Some behavioral events such as the monkey touching the object or the onset of the object displacement by the monkey were controlled during the experiment, but their online-detected timing was approximate and not saved (see details in section Sec. ‘Behavioral control system’). However, these events can be relevant for data analysis and they were thus computed offline from the analog signals of the four FSR sensors measuring the monkey’s grip and the HE sensor measuring the object displacement. We implemented a custom-made Matlab Event-Detection toolbox to detect 8 specific events: the precise timing of object touch (OT) and object release (OR) from the force traces, as well as the timing of displacement onset (DO) and object back to baseline (OBB) from the displacement trace, and finally the onset and offset of the plateau phase in the force and displacement traces. The plateau phase of the displacement signal indicates the timing and stability of the holding period, and its onset is used to calculate offline the hold start (HS) signal. The toolbox performed an automatic detection of these events and their timing was first approximated by threshold crossing and then fine-tuned by further comparison of the traces with baseline level before the point of threshold crossing. Since the automatic detection was prone to errors, the trials were visually inspected one by one and the timing of the automatically detected events were manually corrected if they did not match the event times as visually identified. In addition, a Matlab script was used to inspect the load force traces in each trial to control if the actual object load corresponded to the programmed object load. This procedure ensured that the electromagnet controlling the object load was properly activated throughout the recording session.

### Offline spike sorting

The spike waveforms which were extracted and saved (in the nev file) during the recording were offline sorted using the Plexon Offline Sorter (version 3.3.3). To keep the variability in the half-manual spike sorting at a minimum, all sortings were performed by the same person (A. Riehle). The spike sorting started with loading the complete nev file of a session into the Plexon Offline Sorter. The spike sorting was performed on a duplicate of the data file to keep the original data intact. We started by merging all online extracted waveforms back into one pool per channel and marking them as “unsorted waveforms” in the Plexon Offline Sorter. Intentionally, we ignored here the results of the preliminary waveform sorting (units 0-16 in the nev file) that were performed during the recording via Central Suite software, because this procedure served solely as an online quality overview of the spiking activity. For the invalidation of cross-channel artifacts (e.g., chewing artifacts) all waveforms that occurred simultaneously on a defined percentage of channels (70%) were then marked as “invalidated waveforms” in the Plexon Offline Sorter. Such artifacts occurred only in the recording session of monkey L. Furthermore, a waveform rejection was performed. Thereby all waveforms of abnormally large amplitude and/or atypical shape on a channel were manually also marked as “invalidated waveforms” in the Plexon Offline Sorter.

The actual spike sorting was then performed on the remaining unsorted waveforms (i.e., those not marked as invalidated waveforms) individually for each channel. We used different algorithms to split these waveforms into clusters in a 2- or 3-dimensional principal component (PC) space. The dimensionality of the PC space was chosen according to the best separation. The main algorithms used were K-Means(-Scan) and Valley Seeking (chosen according to the best separation). We used a fixed threshold for outliers (a parameter to be determined in the Plexon Offline Sorter) between 1.8 (K-Means) and 2 (Valley Seeking) to get comparable sorting results. The spikes of the sorted clusters were then controlled using the inter-spike interval (ISI) distributions and the auto- and cross-correlation plots. Units were ordered manually from best to worst (assigning increasing unit IDs 1-16 in the Plexon Offline Sorter) by considering the amplitude of the waveform (the higher the better), the outcomes of the ISI analysis (no or low number of spikes with an ISI smaller than 2 ms), the correlation histograms, and identifiable cluster shapes. Waveforms in the cluster with the highest unit ID (worst) on a given channel may contain multi-unit activity. Clusters with unacceptable outcomes (completely or partly overlapping waveforms), including those with only a few spikes, were left assigned as “unsorted waveforms” in the Plexon Offline Sorter. This offline spike sorted nev file was saved under the file name of the original nev file with an added two-digit numeric postfix (e.g. −01). In this file, unit ID 255 contains invalidated waveforms, unit ID 0 contains the unsorted waveforms (that may enter a further cluster analysis for spike sorting), and unit IDs 1-16 contain the time stamps and waveforms of the sorted single- or multi-units (as in the Plexon Offline Sorter). Unit IDs that are considered to represent multi-unit activity (MUA) as opposed to single-unit activity (SUA) are documented in the metadata. The nev file with the sorted units can be loaded again into the Plexon Offline Sorter to visualize all the sorted spikes and rework the spike sorting.

### Summary of setup differences

Even though overall the same setup was used for both monkeys, we highlighted above several differences that are important to keep in mind (marked in yellow and red for monkey L and N, respectively, in the referenced figures). The differences are summarized below:

- The electrode configurations of the array are not identical between the two monkeys (cf. Fig. 1).
- As headstage, the Samtec or Patient Cable was used for monkey L and N, respectively (cf. Figs 3 and 4).
- The software version of Central Suite was updated between the recording periods of the monkeys. This led to differences in the data formats of the neural raw data (ns5 in monkey L vs. ns6 in monkey N) and in the content of the ns2 file which either only contained the behavioral signals from the target object manipulation (monkey L) or also included a downsampled and filtered version of the neural data (monkey N).
- The settings of the waveform window for the online spike shape extraction in Central Suite are not identical between the monkeys (cf. Fig. 4).
- The LabView program to control and monitor the task and behavior was updated between the monkeys. This led to differences in the binary codings of the digital events stored in the nev file (cf. Table 2).

### Code availability

All available code required to access the data as described in Sec. ‘Usage Notes’ is stored along with the datasets in the data repository (Data Citation 1). The provided code includes, in particular: (i) a snapshot of the Python Neo package (RRID:SCR_000634), (ii) a snapshot of the Python odML package, (iii) the custom-written ReachGraspIO extending the Neo package, (iv) the example script described in Sec. ‘Usage Notes’, (v) code to reproduce Figs. 5, 6, 7, and 8, and (vi) the code demonstrating how to access the data in saved Matlab files (additionally made available for convenience). A static version of the Electrophysiology Analysis Toolkit (Elephant) package (RRID:SCR_003833) is provided for demonstration purposes in the example script, but is not required to load the data.

In addition to these frozen versions of the code, we recommend to use updated versions of the code to benefit from future enhancements, bug fixes and increased compatibility with future Python releases or novel applications that rely on recent versions of Neo and/or odML. Complete link collections to the python-neo and python-odML libraries can be found at http://neuralensemble.org/neo and http://www.g-node.org/projects/odml, respectively. Importantly, both projects are hosted and version-controlled via Github at https://github.com/NeuralEnsemble/python-neo and https://github.com/G-Node/python-odml. Likewise, the Elephant library is hosted at http://neuralensemble.org/elephant and developed at https://github.com/NeuralEnsemble/elephant. Updated versions of the ReachGraspIO and all example code can be found on-line (https://web.gin.g-node.org/INT/multielectrode_grasp).

## Data Records

All data and metadata are publicly available via the G-Node Infrastructure (gin, https://web.gin.g-node.org/) provided by the the German Neuroinformatics Node of the International Neuroinformatics Coordination Facility (INCF). We provide the precise version of the data described here (Data Citation 1), and in addition a repository (https://web.gin.g-node.org/INT/multielectrode_grasp) containing possible future updates of the data and code. Table 3 provides an overview of the name, size, and content of all files for each published dataset of monkey L and N. The datasets of both monkeys consist of three parts: (i) the primary data are provided as the original data files obtained from the Central Suite software stored in the data format specified by the manufacturer (in particular, nev, ns5 and ns6 format) of the neural recording platform, Blackrock Microsystems; (ii) an offline sorted version of the neural spike data (cf. Sec. Offline spike sorting) is provided in a second nev file; (iii) metadata are provided as one file per dataset in the odML format^8,14^.

Alternatively, the folder datasets_matlab contains a set of mat files containing the continuous neural raw data together with the offline sorted spike data, both annotated with the corresponding metadata. The latter is derived from the original data files and is provided for convenience only. Where possible, we suggest to work on the original data files.

### Dataset information

The published recordings were conducted on Friday, December 10, 2010, and Thursday, July 3, 2014, for monkey L and N, respectively (cf. Table 4). For monkey L, this recording day lasted for nearly one hour and a half in which the dataset l101210-001 was the first of 9 recording sessions. For monkey N, the recording day lasted for one hour with the dataset i140703-001 as first out of 3 recording sessions. The datasets of both monkeys were recorded in the late morning. The recording session of monkey L and N lasted 11:49 and 16:43 min in which they performed 204 and 160 trials, respectively. However, monkey L performed only 70% of all trials correctly, whereas monkey N successfully completed 90% of all trials during the recording (cf. Table 5). Nonetheless, the high percentage of error trials in monkey L are mainly caused by too early movement onsets reflecting the eagerness, but also the nervousness of monkey L’s character (cf. Sec. ‘Monkey L’). In contrast to these error types, monkey L used only 12 times the wrong grip compared to monkey N who performed an incorrect grip type 16 times during the session.

For both monkeys the trial types alternated randomly between trials leading to slightly different numbers of trials with the same trial type in each dataset (cf. Table 5).

The quality of the spiking activity in the datasets of both monkeys was high, which allowed us to perform a relatively robust offline spike sorting with high numbers of single unit activity (SUA) distributed over all electrodes of the array (for details see Table 6). For details on how the offline sorting was performed and checked, see Sec. Offline spike sorting and Sec. Spike data quality.

### Information on the metadata framework

All metadata information about the experiment, the subject, the setup, the settings, and the processing of the data were originally distributed over several source files, but were collected offline in one metadata file per recording using the odML metadata framework. The odML framework^14^ is a metadata management project supported by the G-Node (http://www.g-node.org/projects/odml). odML files are machine readable and can be used via application programming interfaces (APIs) in Java (https://github.com/G-Node/odml-java-lib), Python (https://github.com/G-Node/python-odml) and Matlab (https://github.com/G-Node/matlab-odml). Moreover, odML files are human readable and can be screened best by either using the odML Editor which is part of the odML Python API, by converting the file to a spreadsheet using odML-tables (https://github.com/INM-6/python-odmltables), or by viewing the file in a web browser via the metadata stylesheet available as download on the G-Node project website. For details on how to manage metadata for such a complex experiment using the odML framework please see ref. 8. This reference also includes tutorial like code examples on how to use odML files in Python and Matlab (see main article as well as supplementary material).

## Technical Validation

In addition to the preprocessing steps that needed to be performed to gain more content of the raw data, some technical validations of the data also had to be conducted. These technical validations include the correction of the alignment of the data files of the Cerebus DAQ system and a general quality assessment of the data. In order to validate the quality of the recording, a series of algorithms were applied to the data. On the one hand the quality of the LFP signals was assessed per electrode and per trial by evaluating the variance of the corresponding signal in multiple frequency bands. On the other hand the quality of the offline sorted single units (Sec. Offline spike sorting) was determined by a signal-to-noise measure. In addition, noise artifacts occurring simultaneously in the recorded spiking activity were detected and marked. In the following, we explain these technical validation steps in detail.

### Correction of data alignment

The ns6 file starts always 82 samples later than ns2 and nev files. This misalignment is caused by an error in the Blackrock recording software. However, this shift is correctly documented in the header of the ns6 file, and therefore will be automatically corrected in the generic Neo loading routine (cf., BlackrockIO in Sec. ‘Usage Notes’ below). In addition, due to the online filter procedure, the LFP signals in the ns2 file are delayed by approximately 3.6 ms with respect to the time stamps in the nev file and the analog signal of the ns6 file. This offset was heuristically determined, documented in the metadata file, and can be automatically corrected for by the experiment-specific loading routine (cf., ReachGraspIO in Sec. ‘Usage Notes’ below). Note that the time stamps of the spike times provided in the nev file correspond to the start of the waveform, defined as 10 samples before the time point of threshold crossing (left sweep).

### Quality assessment

The occurrence of noise in electrophysiological recordings is to a certain degree unavoidable and therefore needs to be carefully examined. It depends to a large extent on the quality of the headstage used to record the neurophysiological data. In our data, two different types of headstages were used for the two monkeys: the Samtec-CerePort headstage (monkey L) and the Patient Cable (monkey N). The former is much more sensitive to noise than the latter. The type of noise, its cause and appearance in the data is quite variable. Depending on the direct influence of the different types of noise on subsequent analysis methods, one needs to balance the corresponding data rejection between being very permissive and very conservative. For this reason, it is wise not remove or delete data of bad quality, but instead mark them with the judgment of a corresponding quality assessment procedure. For the datasets considered here, we provide the results of our quality assessment of the electrodes, trials and spiking units along with the analysis parameters of the used procedure in the odML metadata files for each recording. The experiment-specific IO integrates this information by annotating the corresponding Neo data objects. This approach not only allows the user to finally decide which data to reject for an analysis, but also provides the opportunity to store simultaneously different quality assessments of the same electrode, trial and unit. This is helpful if one considers that certain types of noise can differently contaminate signals in different frequency bands. The quality of the recorded signals was therefore separately tested for the sorted spike data and different frequency bands of the LFP data. The used corresponding procedures are described in detail below.

### LFP data quality

The LFP data were examined for noise in three broad frequency bands excluding the 50 Hz European line noise (low: 3 Hz-10 Hz, middle: 12 Hz-40 Hz, high: 60 Hz-250 Hz) in each session individually. The goal of the quality assessment was, first, to detect channels with a noisy signal throughout the session and, second, to detect noisy trials in the remaining “clean” channels. To do so, the analog signals of each electrode were first z-scored and filtered in the three frequency bands (low, middle, and high) using a Butterworth filter (of order 2, 3, and 4, respectively). For each frequency band the quality assessment analysis was carried out separately. The detection of noisy electrodes was performed in three steps:

- **Step 1** The variance of the filtered analog signal of each electrode was calculated over the complete session.
- **Step 2** Out of the 96 resulting variance values, outliers were identified as those values outside a user-defined range. The range was defined as follows: (i) values between a lower (e.g., 25th) and an upper (e.g., 75th) percentile (*L* and *U*), (ii) the range of acceptable values was defined by
  (1)[L−w⋅(U−L),U+w⋅(U−L)],
  where *w* is a user-defined whisker coefficient (e.g., *w*=3).
- **Step 3** The analog signals classified as outliers in step 2 were visually controlled by comparing them to the analog signal of an electrode with a typical variance value. If the results were either too conservative or too permissive, the detection procedure was repeated by manually adapting the chosen parameters (*L*, *U*, and *w*), correspondingly.

The electrode IDs of the final outliers as well as the parameters chosen for their detection were saved in the odML metadata file of the corresponding recording and marked as *noisy* for the tested frequency band.

For the remaining non-noisy electrodes, an analogous procedure was carried out afterwards to detect noisy trials. The procedure differed in one respect: the variance of the filtered analog signal was calculated for each trial on each electrode separately. At the end, the trial IDs of the identified outliers were pooled and marked as noisy for the tested frequency band on all electrodes. The marked trial IDs were saved in the odML metadata file of the corresponding recording together with the chosen analysis parameters for their detection. Note again that with this procedure a trial is marked as noisy on all electrodes as soon as it is classified as noisy on one electrode.

### Spike data quality

To test and judge the quality of the spike data, the results of the offline spike sorting were controlled first, for the signal-to-noise ratio (SNR) from the waveforms of the identified single units and second, for the occurrence of hyper-synchronous event artifacts.

1. To calculate the SNR for each identified unit in the sorting results a method introduced used by ref. 15. The SNR was defined as the amplitude (*A*, trough-to-peak) of the mean waveform (<*w*>) divided by twice the standard deviation of the waveform noise (*SD*_*noise*_) of unit *u*:
  (2)SNRu=A<w>/(SDnoise⋅2),
  where *SD*_*noise*_ was computed by averaging the standard deviations (SDs) obtained from each sample point across the original waveforms (SD of the waveform noise adapted refs. 16 and 17). For all identified single units in the datasets published here, the determined SNRs ranged between 1.5 and 12. Corresponding ref. 17 the quality of the spike sorting of an identified unit is good if the SNR is above 4, is fair if the SNR ranges between 2 and 4, and is poor if the SNR ranges between 1 and 2. Units with an SNR below 1 are not considered as signals. For a conservative analysis of the spike datasets, we recommend to use only single units with a SNR of 2.5 or higher, which was our choice, e.g., in ref. 12. The results of the SNR analysis of the performed spike sorting were saved in the odML metadata file of the corresponding recording and units were annotated accordingly.
2. Since correlation analysis of spike data is very sensitive to cross-electrode artifacts which would produce unwanted false positive results, we controlled the sorted spike data on their original time resolution (*δ*=1/30 ms) for potential occurrence of hyper-synchronous event artifacts. For this, we computed the population histogram, i.e. the sum of the spikes across all sorted single units in the dataset in bins of *δ*, and detected if there were entries larger or equal to 2. To our surprise these hyper-synchronous spikes, which are likely to be attributed to cross-channel noise, survived the spike sorting including the cross-channel artifact removal by the Plexon Spike sorter. We indeed detected these spike artifacts during a preliminary analysis of a previous study^12^. The number of single units participating in these events ranged from 2 to over 30 and a statistical analysis showed that the frequency of their occurrence largely exceeded the expected value considering the observed population firing rate. Furthermore, a *δ*-binned time histogram of the population spiking activity triggered around the occurrence times of the hyper-synchronous events revealed also increased spiking activity in the preceding or following bin of the event. For a conservative analysis of the spike datasets, we recommend to treat the spikes participating in a hyper-synchronous event as well as the spikes occurring within a short time interval around this event (±1*δ*) as artifacts of unknown origin and to remove them subsequently before performing any analysis of the spike data.

To illustrate these points, in ref. 12 we combined both quality assessments of the spike data and only considered spikes with a *SNR*>2.5 and additionally removed all hyper-synchronous events with≥2 spikes.

## Usage Notes

In the following, we describe how the provided data files (Sec. Data records) can be practically used in a data analysis scenario. To this end, we first briefly present the open source software libraries we recommend to use in order to access data and metadata using the Python programming language. We also demonstrate how to merge data and metadata in a common representation that facilitates data handling and analysis. Finally, we present an example program that produces a visualization of the most important data items contained in the files, and that can be used as a template script for accessing the provided data. All software discussed below is provided with the datasets, and a link to the repository is listed in Sec. Code availability.

### Libraries for Data Access

As outlined above, the datasets are stored in two types of files. The primary data, and the spike sorted data, are provided in the data format (in particular, the nev, ns5 and ns6 format) specified by Blackrock Microsystems, the manufacturer of the recording hardware. Second, metadata are provided as one file in the odML format^14^. While data and metadata are provided in documented file formats (see http://blackrockmicro.com/ and http://www.g-node.org/projects/odml, respectively), the mere knowledge of the highly complex internal structure of the files is insufficient to practically make use of their content. In particular, implementations of corresponding loading routines performed from scratch by individual researchers are likely to be incoherent and error-prone. Thus, in the following we will use two community supported open-source libraries to separately load primary data and metadata into a generic, well-defined data representation.

We chose the data object model provided by the open-source Neo library^18^ as the primary representation of the datasets (http://neuralensemble.org/neo/). Neo provides a hierarchical data structure composed of Python objects that aim to represent electrophysiological data in a generic manner. In addition, Neo provides a number of I/Os that enable the user to read from (and in part, write to) a large number of open and vendor-specific file formats. In particular, Neo provides an I/O module for the file format used by Blackrock Microsystems (class BlackrockIO in file neo.io.blackrockio.py). The output of this I/O is a Neo data structure that is a faithful representation of the contents of the primary data files. For detailed information on the structure of the Neo data object model, please consult^18^ and the online documentation (http://neo.readthedocs.io/en/latest/index.html). The specific realization of the Neo output structure generated by the read_block() and read_segment() methods of the BlackrockIO are given in detail in the I/O’s documentation.

Here, we briefly summarize the output of the reach-to-grasp datasets obtained when calling the I/O. The read_block() method of an instantiation of the BlackrockIO returns a Neo Block object as a top level grouping object representing one recording session. In the hierarchy directly below the Block is one single Segment object spanning the complete continuous recording, and one ChannelIndex object for each of the 96 electrodes of the Utah Array (Sec. ‘Surgery and Array Location’) and each of the 6 sensor signals monitoring the target object manipulation (Sec. Experimental apparatus). The data from these 102 recording channels is each saved in one AnalogSignal object. All of these are linked to the Segment and the respective ChannelIndex object. Likewise, the spike times (and optionally, the spike waveforms) of each identified unit are saved to a SpikeTrain object. As for the AnalogSignal objects, these are linked to the Segment, and to the ChannelIndex object via a Unit object. Finally, all digital events are saved into a single Event object that lists their time of occurrences and the corresponding event IDs. Additional information from the file is provided as annotations on each individual Neo object (accessible via the annotation property of the object), in particular as annotations to the top level Block object. Note, that although this generic I/O can be used to access the raw data records, no interpretation of the file contents is given. For example, digital events are not interpreted as behavioral events, but only given as the decimal codes (cf. Table 2).

In order to access the metadata stored in the odML file, we use the corresponding library python-odML described in ref. 14. In short, odML files store metadata in form of hierarchically structured key-value pairs. The odML files accompanying the provided datasets contain extensive metadata grouped into the following eight top-level sections: Project (general information on the reach-to-grasp project), Subject (information on the monkey), Setup (details of the experimental apparatus), Cerebus (settings of the recording system), UtahArray (information on the multi-electrode array including spike sorting results and the corresponding quality assessment), Headstage (general settings), Recording (task settings, trial definitions with event time stamps and periods), PreProcessing (results of LFP quality assessment and general information on the spike sorting procedure). A tutorial on how to work with the odML library can be found in the online documentation shipped with the library (https://g-node.github.io/python-odml/), and a more detailed description of how to manage metadata by example of the odML framework can be found in ref. 8. In short, the library supports to read the content of an odML file, provides an API to navigate through the hierarchical structure, and to extract metadata of interest from the key-value pairs. Thus, the python-odML library provides a standardized way to access stored metadata records.

As a next step, we combine the primary data and metadata in a manner that is specific to this experiment and aids the analysis process. To this end, the relevant metadata that were extracted from the odML are attached as annotations to data objects in the hierarchical Neo structure. For example, metadata information for a particular single unit originating from the spike sorting process may be attached to the Neo objects representing the sorted spike data of that unit. The task of combining the primary data and metadata is performed by a custom-written Python class named ReachGraspIO that is derived as child class from Neo’s BlackrockIO class. For a full documentation of the input arguments, methods, and outputs of this class, please refer to the class documentation in reachgraspio.py. In short, invoking the read_block() method of the ReachGraspIO performs the following steps under the hood: (i) read the primary data using the read_block() method of the parent class (BlackrockIO) as described above, (ii) read the metadata using the python-odML library, (iii) interpret event data based on the digital events (e.g., detect trial start or reward), and (iv) add relevant metadata to the Neo data object using the annotation mechanism. Thus, the Neo Block object returned by the ReachGraspIO contains extensive information attached as annotations of the individual Neo objects, in particular, about whether a SpikeTrain is classified as SUA or MUA, about the spatial positioning of electrodes, or about the identities of electrodes that should be rejected. A full list of these metadata annotations can be found in the documentation of the read_block() method in the file reachgraspio.py.

In summary, for practical purposes, the resulting data structure of the ReachGraspIO hosts a complete representation of the data and a synthesis of the metadata relevant for analysis. This representation may be saved to disk in a standardized container format (e.g., .mat or HDF5), such that the exact same data and metadata context can also be accessed from other programming languages. For illustration, we provide the data object in the Matlab file format (.mat) in the folder datasets_matlab, containing Matlab structs resembling the Python Neo objects.

### Practical Example Code

In the following we demonstrate how to use the ReachGraspIO in practice in order to load and visualize the datasets. We follow the file example.py, which is contained as part of the code included with the published datasets. The goal of this program is to create a figure showing the raw signal, LFP, spikes (time stamps and waveforms), and events in a time window (referred to as analysis epoch) around TS-ON of trial 1 for electrode ID 62.

In a first step, we load the data using the ReachGraspIO. Considering that only for monkey N an online filtered version of the LFP data is available in the ns2 file, in the following we calculate offline an LFP signal from all raw signals contained in the ns5 or ns6 files using a non-causal low-pass Butterworth filter implemented in the Electrophysiology Analysis Toolkit (Elephant; http://neuralensemble.org/elephant/), which provides analysis capabilities for data stored in the Neo representation. The parameters of this filter are chosen identical to those of the causal filter for the LFP recorded online in monkey N (Sec. Neural recording platform).

In a subsequent step, we extract all TS-ON events in correctly performed trials. To this end, we use the function get_events() contained in the utility module neo_utils.py. The function extracts a list of events contained in one Event object of the loaded Neo Block given the filter criteria specified by the parameter event_prop. In our example, the used filter criteria select all events from the Event object “TrialEvents” with a trial_event_labels annotation set to TS-ON, and a performance_in_trial annotation indicating a correct trial.

In a next step, we create Epoch objects representing analysis epochs around the extracted TS-ON events. To this end, we use add_epoch() also contained in the utility module neo_utils.py. The function excepts the previously extracted TS-ON events as trigger, and defines epochs of a given duration around this trigger. The resulting Epoch object is called “analysis_epochs”.

Next, we cut the data according to the analysis epochs and align the cutouts in time. This operation is performed by cut_segment_by_epoch(), which returns a list of Segment objects, each containing data of one analysis epoch. The Segments are annotated by the corresponding annotations of the Neo Epoch. In addition, the list of Segment objects is grouped in a new Neo Block, named “data_cut_to_analysis_epochs”. This representation now enables the analysis of the data across trials in the defined analysis epochs.

In our example, we show how to create a plot of the data of the analysis epoch in one behavioral trial on the selected electrode. To select the Neo Segment corresponding to the first correct behavioral trial from the Block of the cut data obtained in the previous step, we apply the Neo filter() function.

From the selected Segment, LFP data and raw signals can be obtained via the AnalogSignal objects referenced by the analogsignals property, while spike trains and corresponding unit waveforms can be extracted from the SpikeTrain objects referenced by the spiketrains property. The remainder of example.py uses the matplotlib library to create a figure of the data.

All data and metadata files as well as the code described above can be found in the data repository (Data Citation 1 and https://web.gin.g-node.org/INT/multielectrode_grasp). The subdirectory datasets contains all data files and the metadata odml file for the two provided recording sessions. The subdirectory code contains the files example.py and neo_utils.py. For further reference and inspiration this subdirectory also contains the Python scripts generating the data figures in Figs 5,6,7 and 8. Furthermore, the subdirectories to code contain frozen versions of the required libraries (python-neo, python-odml, elephant) as well as the custom loading routine combining data and metadata (reachgraspio.py). Finally, the datasets_matlab directory contains the annotated Neo data object containing all primary data saved in the mat file format. Updated versions of the data and code, e.g., adapted to new releases of the Neo library, will be made available in the same data repository.

## Additional information

**How to cite this article**: Brochier T. *et al.* Massively parallel recordings in macaque motor cortex during an instructed delayed reach-to-grasp task. *Sci. Data* 5:180055 doi: 10.1038/sdata.2018.55 (2018).

**Publisher’s note**: Springer Nature remains neutral with regard to jurisdictional claims in published maps and institutional affiliations.

## Supplementary Material



## Figures and Tables

**Table 1:** Overview of the six object sensors used to monitor and control the monkey’s behavior.

sensor	channel ID	label	located at	activated by	used to identify
FSR 1	137	GF pr1	object’s top	index finger’s touch	PG type
FSR 2	138	GF side1	object’s left	middle finger’s touch	SG type
FSR 3	139	GF pr2	object’s bottom	thumb touch	PG type
FSR 4	140	GF side2	object’s right	thumb touch	SG type
FSR 5	141	LoadForce	object’s spring	object loading	pulling force
HE	143	Displ	object’s shuttle	object displacement	object’s position
The first four force sensitive resistance (FSR) sensors are used to monitor the applied grip type. They are located on the surface of each object side and are activated by the touch of the monkey’s finger. The fifth FSR is located at the spring counterbalancing the pull resistance of the object and is used to measure the pulling force applied by the monkey. The hall-effect (HE) sensor is located along the low-friction shuttle of the object and used to measure the position of the object. The signals of all sensors are saved in the ns2 file with the stated channel ID and label (cf. Fig. 4).					

**Table 2:** Translation table of 8-bit binary to decimal event codes and their interpretation in a trial context.

**decimal code**	**(8-bit) binary code**								trial interpretation		
	bit 7	bit 6	bit 5	bit 4	bit 3	bit 2	bit 1	bit 0			
**65296**	0	0	0	1	0	0	0	0	**TS-ON**	(L,N)	
**65280**	0	0	0	0	0	0	0	0	**TS-OFF**	(L)	
**65344**	0	1	0	0	0	0	0	0	**WS-ON**	(L)	
**65360**	0	1	0	1	0	0	0	0	(N)		
**65349**	0	1	0	0	0	1	0	1	**PG-ON**	**(CUE-ON)**	(L)
**65365**	0	1	0	1	0	1	0	1			(N)
**65354**	0	1	0	0	1	0	1	0	**SG-ON**		(L)
**65370**	0	1	0	1	1	0	1	0			(N)
**65344**	0	1	0	0	0	0	0	0	**CUE-OFF**	(L)	
**65360**	0	1	0	1	0	0	0	0	(N)		
**65353**	0	1	0	0	1	0	0	1	**LF-ON**	**(GO-ON)**	(L)
**65369**	0	1	0	1	1	0	0	1			(N)
**65350**	0	1	0	0	0	1	1	0	**HF-ON**		(L)
**65366**	0	1	0	1	0	1	1	0			(N)
**65385**	0	1	1	0	1	0	0	1	**SR**	(+ LF)	(L,N)
**65382**	0	1	1	0	0	1	1	0		(+ HF)	(L,N)
**65509**	1	1	1	0	0	1	0	1	**RW-ON**	(+ CONF-PG)	(L,N)
**65514**	1	1	1	0	1	0	1	0		(+ CONF-SG)	(L,N)
**65376**	0	1	1	0	0	0	0	0	**GO-OFF/RW-OFF**	(L,N)	
**65312**	0	0	1	0	0	0	0	0	**STOP**	(L,N)	
**65280**	0	0	0	0	0	0	0	0		(L)	
**65391**	0	1	1	0	1	1	1	1	**ERROR**	(+ switch)	(L)
**65359**	0	1	0	0	1	1	1	1			(L)
	**pump**	**LED c**	**switch**	**TS**	**LED b,l**	**LED t,r**	**LED t,l**	**LED b,r**			
The 8-bit binary event code created by LabView states the activation (bit status 1) and deactivation (bit status 0) of the LEDs of the cue system (c:center, t:top, b:bottom, l:left, r:right), the table switch (switch), the reward pump (pump) or the trial start (TS) internally set by LabView. During each trial the (8-bit binary) status of these devices/settings (cf. bottom row) were sent from LabView to the NSP of the Cerebus DAQ system (Figure 2). There, the event codes were converted to a decimal code of the bit sequence assuming another byte with all bits set to 1 in front. The decimal event codes are found in the nev files with a time stamp. The correct interpretation of these events in context of a trial are here indicated in the second column from the right. Due to different versions of the LabView control program for monkey L and N the decimal codes for the same event may differ between the monkeys (cf. first column from the right). Some event codes (65381, 65386, 65390, 65440, 65504) are not listed here, because they do not have a concrete meaning and occur only sporadically in the nev file due to a mistake in the sampling of the digital events - they have to be ignored. Except for the "ERROR" codes, the event codes are sorted in sequential order from top to bottom with respect to the task, i.e. their order corresponds to the sequence found in the nev file in a reach-to-grasp trial. Note that some events are represented by the same decimal codes and are just differently interpreted due to their sequential occurrence in a trial (cf. TS-OFF and STOP, as well as WS-ON and CUE-OFF).											

**Table 3:** Overview of files (names, size, and content) for each provided dataset of monkey L and N.

**monkey L**		
**file names**	**file size**	**file content**
l101210-001.ccf	108.2 kB	cerebus configuration file
l101210-001.nev	287.7 MB	digital events, unsorted spikes times & waveforms
l101210-001-02.nev	287.7 MB	digital events, sorted spikes times & waveforms
l101210-001.ns2	8.5 MB	analog signals of object sensors
l101210-001.ns5	4.1 GB	raw neuronal signal
l101210-001.odml	2.7 MB	metadata
**monkey N**		
**file names**	**file size**	**file content**
i140703-001.ccf	187.1 kB	cerebus configuration file
i140703-001.nev	168.3 MB	digital events, unsorted spikes times & waveforms
i140703-001-03.nev	168.3 MB	digital events, sorted spikes times & waveforms
i140703-001.ns2	204.7 MB	analog signals of object sensors and LFP signals
i140703-001.ns6	5.8 GB	raw neuronal signal
i140703-001.odml	2.3 MB	metadata
Besides the listed data and metadata files, we also provide a mat file collection from the dataset of each monkey that contains the analog and digital signals of the nev, ns5/6, and ns2 files annotated with the corresponding metadata information of the odml file.		

**Table 4:** Overview of recording days of the published datasets.

**monkey**	**date**	**weekday**	**start**	**dur**	**# rec**	dur of rec*-001
**L**	2010-12-10	Friday	10:50 am	01:28 h	9	11:49 min
**N**	2014-07-03	Thursday	10:41 am	00:51 h	3	16:43 min
For both monkeys, we chose to publish the first dataset (rec*-001) of the recording day. For details on the published datasets see Table 3 and Table 5.						

**Table 5:** Overview of trials in the published datasets.

**monkey**	**# trials**	**# error trials**		**# correct trials**				
	**total**	**total**	**grip**	**total**	**SG-LF**	**SG-HF**	**PG-LF**	**PG-HF**
**L**	204	69	12	135	41	30	31	33
**N**	160	19	16	141	35	35	35	36
Of the stated number of error trials, monkey L and N used the wrong grip type in 12 and 16 trials, respectively. In the remaining error trials the monkeys initiated the movement too early. Trial types were altered randomly in the recordings which led to slightly different trial numbers for the different trial types.								

**Table 6:** Overview of offline sorted single and multi unit activity (SUA and MUA).

**monkey**	**sorting ID**	**# SUA**	**# MUA**	**# electrodes with**	
				**SUA**	**SUA or MUA**
**L**	*−02	93	49	65	86
**N**	*−03	156	19	78	89
For the recording of monkey L, it was possible to sort out 93 SUAs distributed over 65 of the 96 electrodes of the Utah array. 28 of the 49 MUA were found on one of these 65 channels containing SUA activity. For the recording of monkey N, it was possible to sort out 156 SUAs distributed over 78 electrodes. Here, 8 of the 19 MUA were found on one of these 78 channels. For details on the offline spike sorting see Sec. Offline spike sorting.					
